# PAMI Syndrome: Two Cases of an Autoinflammatory Disease with an ALPS-Like Phenotype

**DOI:** 10.1007/s10875-022-01265-x

**Published:** 2022-04-19

**Authors:** Fionnuala Cox, Venetia Bigley, Alan Irvine, Ronan Leahy, Niall Conlon

**Affiliations:** 1 Department of Immunology, St. James’s Hospital and Trinity College, Dublin 8, Ireland; 2 Translational and Clinical Research Institute, Newcastle University, Newcastle upon Tyne, UK; 3 Department of Paediatric Dermatology, CHI Crumlin Hospital, Dublin 12, Ireland; 4 Department of Immunology, CHI Crumlin Hospital, Dublin 12, Ireland

To the Editor,

Proline-serine-threonine phosphatase interacting protein-1 (PSTPIP1)–associated myeloid-related inflammatory (PAMI) syndrome with hyperzincemia is a rare autoinflammatory disorder associated with enhanced pyrin activity and inflammasome activation. Clinical features include cutaneous and systemic inflammation, failure to thrive, hepatosplenomegaly, lymphadenopathy, and cytopenias. Pathogenic variants in *PSTPIP1* are frequently associated with pyogenic arthritis, pyoderma gangrenosum, and acne (PAPA) syndrome [1, 2]. Patients with PAMI have distinct mutations (E250K or less commonly E275K), and exhibit more pronounced systemic inflammation and cytopenias but less joint involvement than individuals with PAPA syndrome [3].

We present two unrelated, Caucasian Irish cases of PAMI syndrome and describe the inflammatory signature associated with the condition. Both patients have the same heterozygous E250K variant in *PSTPIP1*; one was diagnosed as an adult, the other in childhood.

## Patient 1

The first patient is a 46-year-old Irish male born to non-consanguineous parents with no family history of note. At 2 years, he developed colitis, failure to thrive, recurrent lower respiratory tract infections, and juvenile idiopathic arthritis (JIA). He was noted to have selective IgA deficiency, with a polyclonal elevated IgG. In adolescence, he developed neutropenia and thrombocytopenia refractory to steroids and ultimately had a splenectomy. Post splenectomy, the platelet count recovered; however, neutropenia persisted. Early adulthood was characterized by non-malignant lymphadenopathy, intermittent synovitis, and nodulo-cystic acne. There were occasional flares of colitis and minor respiratory tract infections. A lymph node biopsy was consistent with reactive changes, follicular hyperplasia, and preserved architecture, while bone marrow aspirate demonstrated a hypercellular marrow. Inflammatory markers were persistently elevated (Table 1). The patient was reviewed at two international centers of excellence during early adulthood in search of clarity on a diagnosis. Next-generation sequencing (NGS, “TIGER Panel”) available at the time was uninformative. In light of clinical feature, an elevated vitamin B_12_ increased double-negative T cells (DNT) and repeated abnormal apoptosis assays (CD95L induced apoptosis assay of PHA-treated blastocysts; Table 1); a diagnosis of autoimmune lymphoproliferative syndrome (ALPS) was proposed.

**Table 1:** Laboratory characteristics of patients 1 and 2. Measurements obtained prior to the commencement of biologic therapy. *CD95L induced apoptosis of patient PHA-blastocysts. Our patient demonstrated an apoptosis quotient of 65–70%, compared with a travel control. The assay is performed over a concentration range and normal activity should be > 80%

Laboratory characteristics	Patient 1	Patient 2
**Biochemistry**		
Serum zinc (μmol/L)	185	88 (9–22)
Serum calprotectin (ng/mL)	Not measured	385 (26–78)
LDH (U/L), pre-treatment	453 (135–250)	1389 (325–670)
Ferritin (ng/mL), pre-treatment	340	1250–2152 (21–274)
CRP (mg/L), pre-treatment	42–95	34–94 (< 5)
Serum amyloid A (mg/L), pre-treatment	22–140	6.4–222 (< 5)
C3 (g/L)	1.46	1.94 (0.7–1.7)
C4 (g/L)	0.26	0.29 (0.09–0.4)
Serum vitamin B_12_ (pg/mL)	1277	1178 (197–866)
Serum Fas ligand	Normal	Normal
Apoptosis*	Abnormal	Not measured
**Serology**		
IgG (g/L)	21.6	12 (3–9)
IgA (g/L)	< 0.05	0.96 (0.15–0.7)
IgM (g/L)	0.7	2.3 (0.4–1.6)
Anti-nuclear antibody	Negative	Negative
Anti-neutrophil antibody	Negative	Negative
Rheumatoid factor	Negative	Negative
**Lymphocyte subsets (values in cells/μL)**		
T cells (CD3 +)	1980 (797–2996)	1568 (700–4200)
CD4 + T cells	889 (502–1749)	947 (300–2000)
CD8 + T cells	757 (263–1137)	527 (300–1800)
TCRαß + T cells (%)	83	89
TCR γδ + T cells (%)	10	11
Double-negative T cells (% CD3 + cells)	7	Not detected
B cells (CD19 +)	422 (99–618)	908 (200–1200)
**Plasma cytokines (pg/mL)**		
IL-1β	407	2.6 (0.00–1.26)
ASC	146.7	17.24 (0–0.2)
TNFα	24.3	19.5 (0.00–22.59)
IL-8	36.7 (0.00–33.01)	Not detected
IL-6	37.9	40.4 (0.09–7.26)
IFNγ	27.2	Not detected

Further phenotyping of the DNT population was not typical of ALPS (CD45RA − , CD38 +) and ALPS-associated mutations were not identified by targeted testing. At 44 years of age, a more comprehensive NGS panel for primary immunodeficiency and autoinflammatory conditions (“Invitae”) identified a heterozygous pathogenic variant (E250K) in the *PSTPIP1* gene*.* Plasma IL-1β was grossly elevated in the absence of infection, while levels of IL-6, TNFα, IFNγ, and IL-8 did not differ from controls. Levels of the inflammasome scaffold protein ASC (apoptosis-associated speck-like protein containing a carboxyterminal CARD) were also elevated suggesting activation of the pyrin inflammasome.

Subsequent assessment revealed a marked elevation of serum zinc at 192 μmol/L supporting a diagnosis of PAMI syndrome. Treatment with the IL-1β receptor antagonist anakinra was commenced but an initial response was not maintained. The patient was switched to adalimumab resulting in improvement to musculoskeletal symptoms and lowered inflammatory markers. This has not been sustained long term. Currently, there is consideration to discontinue adalimumab and trial the IL-6 receptor blocker tocilizumab.

## Patient 2

A 15-year-old Caucasian Irish male presented at 6 months of age with prominent splenomegaly and severe anemia. Hemoglobin at presentation was 54 g/L and he required multiple red cell transfusions. Neutropenia and thrombocytopenia were also noted. He suffered recurrent respiratory tract infections, diarrhea, and lymphadenopathy. Anemia improved after a course of oral corticosteroids, but neutropenia, lymphadenopathy, and splenomegaly persisted. In addition, he developed painful polyarthropathy from the age of 2 years, along with *alopecia areata*, and an acneiform rash on his face and dorsum of his hands. Repeated bone marrow aspirates showed a hypercellular marrow with trilineage hematopoiesis, left shift myelopoiesis, lymphocytosis, and evidence of patchy reticulin fibrosis on staining. No lineage dysplasia was seen. LDH was persistently elevated, as were inflammatory markers (ESR, CRP, serum amyloid A; Table 1). Serum ferritin was high at 1000–2000 ng/mL.

Lymphocyte immunophenotyping demonstrated a mild increase in DNT population (1.5%), along with a relative increase in the proportion of γδ_-_T cells (11%). The T cell TCRvβ repertoire was normal as were T cell proliferation studies, apoptosis studies, and expression of SAP, XIAP, and FOXP3. Vitamin B_12_ was raised, and serum Fas ligand was normal, as was sequencing of genes associated with myelofibrosis and neutropenia.

There was a strong family history of autoimmunity; the patient’s mother having a diagnosis of arthritis and sister had *alopecia areata*. There was no consanguinity. Ultimately, a diagnosis of PAMI syndrome was made at the age of 8 years, after whole exome sequencing on neutrophils isolated from the patient and members of his immediate family. He was heterozygous for the E250K mutation in the *PSTPIP1* gene, which was a de novo event; parents did not carry this mutation. A serum zinc measured subsequently was elevated (88 µmol/L).

Adalimumab was commenced shortly thereafter, leading to improved joints and skin symptoms and resolution of lymphadenopathy. Neutropenia has remained unchanged. Plasma ASC was increased (17.24 pg/mL) compared with controls. IL-1β, IL-6, TNFα, IFNγ, and IL-8 were not elevated in plasma.

## Discussion

PAMI syndrome is a recently described clinical entity within a spectrum of *PSTPIP1*-associated inflammatory diseases (PAID) [3]. Hyperzincemia, raised myeloid-related protein (MRP8/14-calprotectin), and elevated inflammatory markers discriminate PAMI syndrome from other PAIDs [3]. Two specific pathogenic variants (E250K, E257K) have been linked to PAMI. From the handful of published case reports, both variants show strong clinical concordance; > 90% have splenomegaly, cytopenias, and systemic inflammation; 80% show non-pyogenic osteoarticular manifestations; and 70% have cutaneous lesions [1]. The systemic inflammatory aspects of PAIDs are a common feature, reflected by elevated CRP and ESR. The data presented here demonstrate increased ASC, a surrogate marker for pyrin inflammasome formation, in both cases.

Our initial investigations focused on identifying an underlying cause for the constellation of splenomegaly and lymphadenopathy in the context of immunodeficiency and autoimmunity, clinical findings not typical of autoinflammatory disease. In light of clinical presentation, non-contributory early genetic studies, and biomarkers (elevated DNT, polyclonal IgG, raised vitamin B_12_, impaired apoptosis), a diagnosis of ALPS-U felt reasonable. Continued observation and improved access to genetic testing lead to a diagnosis of PAMI syndrome.

Our observation of increased baseline lymphocyte counts could indicate an impaired apoptotic pathway, or enhanced cell survival, which underlies the characteristic lymphadenopathy and autoimmune phenotype. Case one demonstrated abnormal apoptosis on repeated assessments, again suggestive of ALPS (Table 1). Whole exome analysis of a pediatric cohort with autoimmunity and lymphoproliferation reminiscent of ALPS identified a single case with the E250K variant of *PSTPIP1* [4].

The pathogenesis of PAMI syndrome has not been fully elucidated. *PSTPIP1* is a cytoskeletal-associated adaptor protein that interacts with pyrin, allowing for the formation and activation of the pyrin inflammasome, which culminates in the caspase-1-dependent processing of inflammatory mediators including IL-1β and IL-18 into their mature form [5]. The oligomerization of the pyrin inflammasome involves an interaction with ASC, a component shared with the NLRP3 inflammasome. It is hypothesized that E250K causes a charge reversal within the *PSTPIP1* protein that increases its proclivity for pyrin, amplifying cytokine release and sequestration of the alarmins MRP-8 and-14 [3]. The elevated levels of ASC identified in both cases are suggestive of baseline activation of the pyrin inflammasome.

Hyperzincemia is pathognomonic for PAMI syndrome, possibly representing a pseudo-elevation, as functional stores are likely depleted due to MRP’s high zinc-binding capacity [5]. Zinc has a key immunomodulatory role in signal transduction and cell-mediated immunity, with deficiency known to result in immune dysfunction. The absence of free-zinc in PAMI syndrome possibly contributes to the associated immunodeficiency profile. There is currently no evidence to use zinc supplementation in these patients.

These cases add from the handful of published case reports to the body of clinical data on PAMI syndrome and illustrates a disease with features of autoinflammation and non-malignant lymphoproliferation. PAMI syndrome should be considered in genetically unidentified cases of ALPS (ALPS-U) or in individuals with incomplete manifestations of the disease. Assessment of serum zinc levels may be a useful and cost-effective preliminary screening test in patients suspected of having this condition. We identified elevated ASC levels in both cases suggesting baseline pyrin activation driving inflammation. Both of our patients demonstrated an initial response to TNF blockade, improved musculoskeletal symptoms, and lower inflammatory markers. Unfortunately, symptom control is waning in patient one and other therapy is being considered; thus, optimum treatment for PAMI is not clear. The determination of the *PSTPIP1* variant ended a 44-year diagnostic odyssey for case one and offered both patients improved therapeutic options and established diagnostic certainty. PAMI syndrome has been diagnosed in acutely unstable neonates, as well as clinically stable adults with indeterminate symptoms, emphasizing the age range at which a definitive diagnosis can be made. As NGS becomes part of routine pediatric and adult care, these cases illustrate the increasing importance of genetic testing and revisiting diagnoses in long-standing patients as new disease entities emerge.

## Data Availability

Please contact the corresponding author Dr. Fionnuala Cox at coxff@tcd.ie if you wish to request further information on the data.
